# A Rare Case of Granular Cell Tumor in the Right Upper Lung of an Adolescent Patient

**DOI:** 10.7759/cureus.21558

**Published:** 2022-01-24

**Authors:** John Grove, Casey Meier, Bahaaeldin Youssef, Patrick Costello

**Affiliations:** 1 Pathology, Lincoln Memorial University-DeBusk College of Osteopathic Medicine, Knoxville, USA; 2 Pathology, East Tennessee State University Quillen College of Medicine, Johnson City, USA

**Keywords:** immunohistochemical, bronchoscopy, lung mass, pediatrics, granular cell tumor

## Abstract

Granular cell tumors (GCTs) are rare neoplasms of neuroectodermal origin characterized by large polygonal cells with abundant eosinophilic and granular cytoplasm. GCTs rarely affect the lungs, with only a few cases reported in the literature. The pathophysiology of this Schwann cell-derived condition is not well understood but is thought to be due to recurring genetic mutations. GCTs have been linked with Noonan syndrome. Here, we report the case of a 17-year-old caucasian male who presented with partial upper airway obstruction due to a GCT. This case promotes awareness among pathologists and clinicians for this condition in the workup of patients presenting with upper airway obstruction.

## Introduction

Granular cell tumors (GCTs) are neoplasms of neuroectodermal origin characterized by large polygonal cells with abundant eosinophilic and granular cytoplasm [1,2]. The underlying pathophysiology of GCTs has been linked to Noonan syndrome with recurrent genetic mutations involving the *PTPN11* gene, *PTEN* gene, or Ras/mitogen-activated protein kinase pathway [1,3,4]. GCTs most commonly occur among adults with a peak incidence between 30 and 50 years of age; however, few cases have been reported in adolescents [2,5,6]. In addition, African American females show a higher incidence with a 5:4 male-to-female ratio [2].

## Case presentation

A 17-year-old caucasian male presented with progressively worsening shortness of breath, exertional dyspnea, and wheezing for four months. His symptoms gradually worsened despite medical treatment with albuterol, ipratropium, montelukast, and loratadine. A pulmonary function test indicated a fixed upper airway obstruction. Chest radiograph and computed tomography were unremarkable. However, a subsequent bronchoscopy revealed a 0.3 cm polypoid mass partially obstructing the right upper bronchus, for which a biopsy was performed.

**Figure 1:**
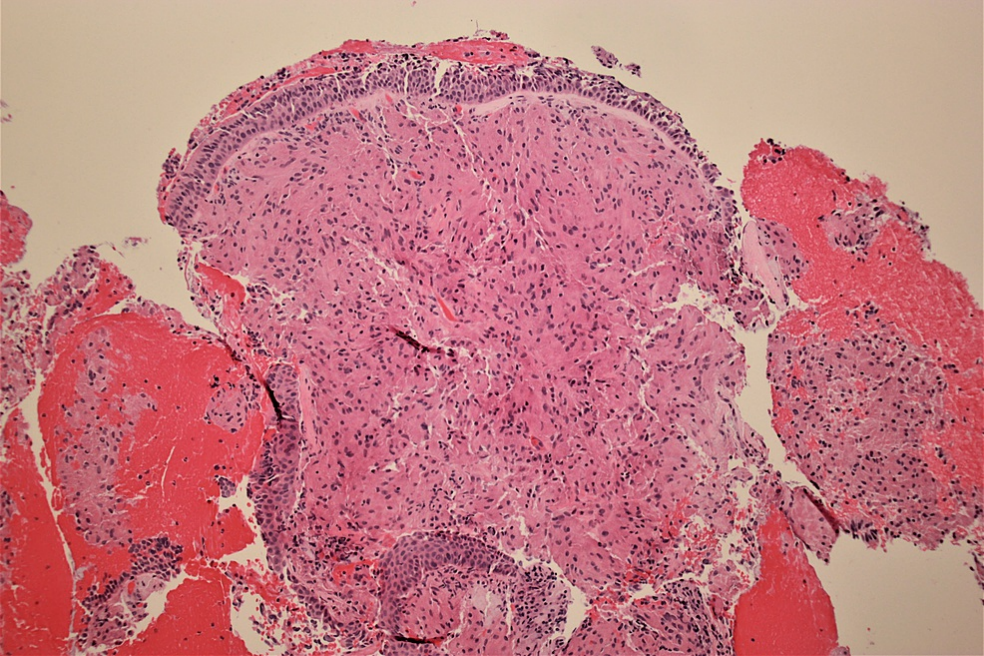
Low-power view of the histologic section revealing sheets of polygonal tumor cells with oval to spindle-shaped nuclei and abundant eosinophilic cytoplasm (hematoxylin and eosin, 40×).

**Figure 2:**
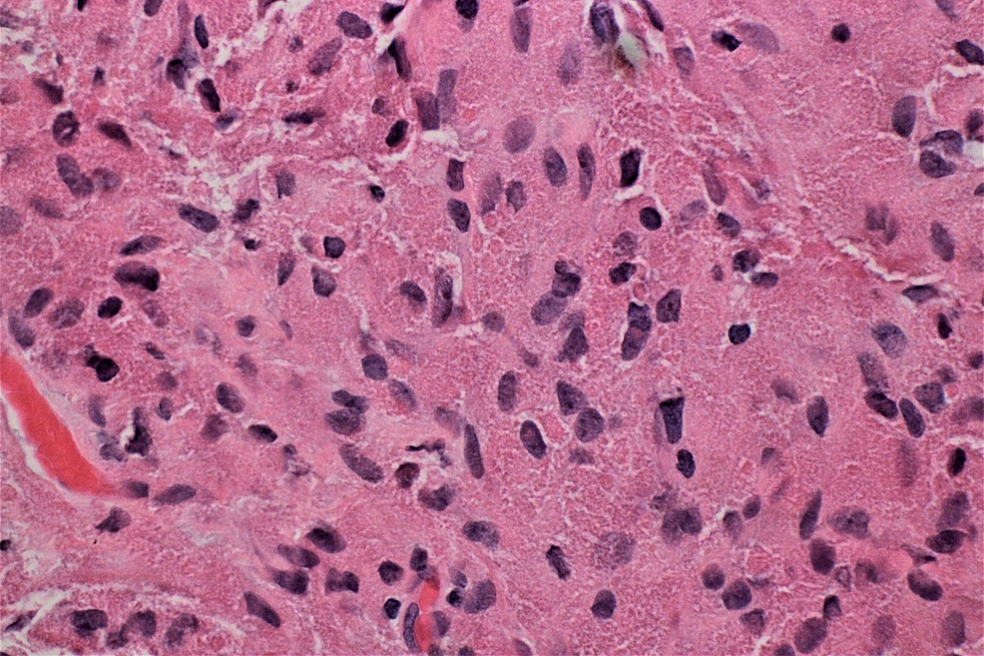
Low-power view of the histologic section revealing sheets of polygonal tumor cells with oval to spindle-shaped nuclei and abundant eosinophilic cytoplasm with coarse granules. Minimal cytologic atypia is identified with absent mitotic figures (hematoxylin and eosin, 100×).

Microscopic examination of the biopsy revealed subepithelial proliferation of bland spindled to polygonal cells with round to oval nuclei and abundant eosinophilic granular cytoplasm (Figures 1, 2). Discrete areas of squamous metaplasia and pseudoepitheliomatous hyperplasia of the overlying respiratory epithelium were present. No overt features of malignancy, necrosis, or abnormal mitotic figures were identified. Immunohistochemical staining showed the neoplastic cells were positive for S100, SOX10, and CD68, and negative for CD163, desmin, keratin AE1/AE3, and Mart1. Histologic and immunohistochemical features supported the diagnosis of a GCT. The patient was lost to follow-up, and his medical management plan was discontinued.

## Discussion

A GCT is a rare soft tissue neoplasm of neuroectodermal origin characterized by large cells with eosinophilic, granular cytoplasm [2]. They predominantly affect adults with a low incidence in children and adolescents. GCTs usually occur in the skin, oropharynx, oral cavity, gastrointestinal tract, or subcutaneous tissue [1]. These tumors rarely occur in the lungs or breasts. Some patients can have multifocal GCTs, which are more likely to recur with a higher possibility of metastasis [7,8]. Immunohistochemical stains are an important diagnostic tool that can help establish the diagnosis of GCTs. Neoplastic cells are usually positive for S100, SOX10, and CD68 [2].

Although the large majority of GCTs are benign, a small portion (1-2%) can be malignant [1,2,5,9]. One study reported a GCT originating in the lung that later metastasized [8]. Other case reports have documented GCTs in the pulmonary system (roughly 80 cases, 20 of which involved the lungs), where the patients’ age ranged from 20 to 57 years [10]. Because the incidence of GCTs is low in general, with few cases reported in the literature, surgical excision of the tumor has been the mainstay of treatment with little information discussed regarding medical management [1,6].

## Conclusions

This unique case report highlights the importance of screening and follow-up to prevent advanced malignancy. It also aims to bring GCTs into attention when considering the differential diagnosis for obstructive airway symptoms. Here, we reported the case of a 17-year-old male presenting with asthma-like symptoms, ultimately leading to the diagnosis of a GCT. Despite the small risk of malignant transformation, performing a thorough clinical evaluation is essential, especially in pediatric patients.
